# The transcription levels and prognostic values of seven proteasome alpha subunits in human cancers

**DOI:** 10.18632/oncotarget.13885

**Published:** 2016-12-10

**Authors:** Yunhai Li, Jing Huang, Jiazheng Sun, Shili Xiang, Dejuan Yang, Xuedong Ying, Mengqi Lu, Hongzhong Li, Guosheng Ren

**Affiliations:** ^1^ Chongqing Key Laboratory of Molecular Oncology and Epigenetics, The First Affiliated Hospital of Chongqing Medical University, Chongqing, China; ^2^ Department of Endocrine and Breast Surgery, The First Affiliated Hospital of Chongqing Medical University, Chongqing, China

**Keywords:** proteasome alpha subunits, cancer, oncomine, TCGA, Kaplan-Meier plotter

## Abstract

Proteasome alpha subunits (PSMAs) have been shown to participate in the malignant progression of human cancers. However, the expression patterns and prognostic values of individual PSMAs remain elusive in most cancers. In the present study, we investigated the mRNA expression levels of seven PSMAs in different kinds of cancers using Oncomine and The Cancer Genome Atlas (TCGA) databases. The prognostic significance of PSMAs was also determined by Kaplan-Meier Plotter and PrognScan databases. Combined with Oncomine and TCGA, the mRNA expression levels of PSMA1-7 were significantly upregulated in breast, lung, gastric, bladder and head and neck cancer compared with normal tissues. Moreover, only PSMA6 and PSMA5 were not overexpressed in colorectal and kidney cancer, respectively. In survival analyses based on Kaplan-Meier Plotter, PSMA1-7 showed significant prognostic values in breast, lung and gastric cancer. Furthermore, potential correlations between PSMAs and survival outcomes were also observed in ovarian cancer, colorectal cancer and melanoma by Kaplan-Meier Plotter and PrognScan. These data indicated that PSMAs might serve as novel biomarkers and potential therapeutic targets for multiple human cancers. However, further studies are needed to explore the detailed biological functions and molecular mechanisms involved in tumor progression.

## INTRODUCTION

Cancer, as a global health problem, accounts for the leading cause of death in most countries and regions, but remains a major challenge in current medicine [1]. In 2016, a total of 1,685,210 new cancer cases and 595,690 cancer deaths are predicted to occur in the United States [2]. Despite improved diagnostics, advanced surgical methods and growing numbers of anti-cancer drugs and targeted therapies, cancer is still a major limitation of patients’ life quality and a severe social and economical burden. It is thus imperative to investigate the underlying mechanisms of cancer initiation and progression, as well as to identify potential biomarkers for improving diagnosis, therapy and prognosis.

The 26S proteasome, consisting of 20S proteasome core and 19S regulatory particles, is a multi-subunit complex playing a central role in degrading obsolete and impaired endogenous proteins [3]. Emerging evidence has indicated that multiple subunits of proteasome were strongly implicated in regulating the biological progression of cancer cells such as proliferation, apoptosis, cell cycle, DNA repair, invasion and metastasis [4–7]. Aberration and abnormal expression of proteasome subunits have been demonstrated in many tumors including breast cancer [8], lung cancer [5, 7], hepatocellular carcinoma [9] and colorectal cancer [10]. For instance, PSMB4, a subunit of the 20S core complex, has been shown to be upregulated in epithelial ovarian cancer, and overexpression of PSMB4 was significantly related to clinicopathological characteristics and worse prognosis in epithelial ovarian cancer patients [11]. Another well studied oncoprotein PSMD10 is frequently overexpressed in hepatocellular carcinoma (HCC) and regulates the balance between apoptosis and cell cycle via the degradation of RB1 and TP53 [12, 13]. Overexpression of PSMD10 promotes HCC invasiveness and metastasis, and could serve as a valuable biomarker for recurrence and survival [9].

Proteasome alpha subunits (PSMAs) are major components of the 20S proteasome core complex. Two rings formed by alpha subunits are necessary for proteasome assembly and the binding of the 19S or 11S regulatory complex [14]. There are seven unique alpha subunits, PSMA1-7, of which several have been demonstrated to be closely associated with cancers. A previous study reported that the mRNA expression of PSMA1 and PSMA5 were significantly increased in pulmonary neuroendocrine tumors compared to normal tissues [5]. Polymorphisms in PSMA4 contribute to lung cancer susceptibility, and upregulated PSMA4 in lung cancer plays an important role in regulating cell proliferation and apoptosis [15, 16]. PSMA7 participates in the degradation of multiple proteins that are necessary for the replication of hepatitis B virus, which is closely related to the development of HCC [17, 18]. In addition, the expression of PSMA7 has been shown to be overexpressed in colorectal cancer and was significantly associated with prognosis in cancer patients [19]. Depletion of PSMA7 in colorectal cancer cells had an inhibition effect on cell invasion and migration [4]. These preliminary studies suggest that PSMAs are involved in multiple human cancers, but a comprehensive analysis of the seven genes, which might act as potential therapeutic targets or prognostic biomarkers, is still absent.

In the present study, we investigated the mRNA expression differences between tumor and normal tissues in multiple cancers for PSMA1-7 using Oncomine and TCGA databases. Additionally, the prognostic significance of these PSMAs was also determined via Kaplan-Meier Plotter (KM Plotter) and PrognScan databases.

## RESULTS

### The mRNA expression patterns of PSMAs in human cancers

**Figure 1:**
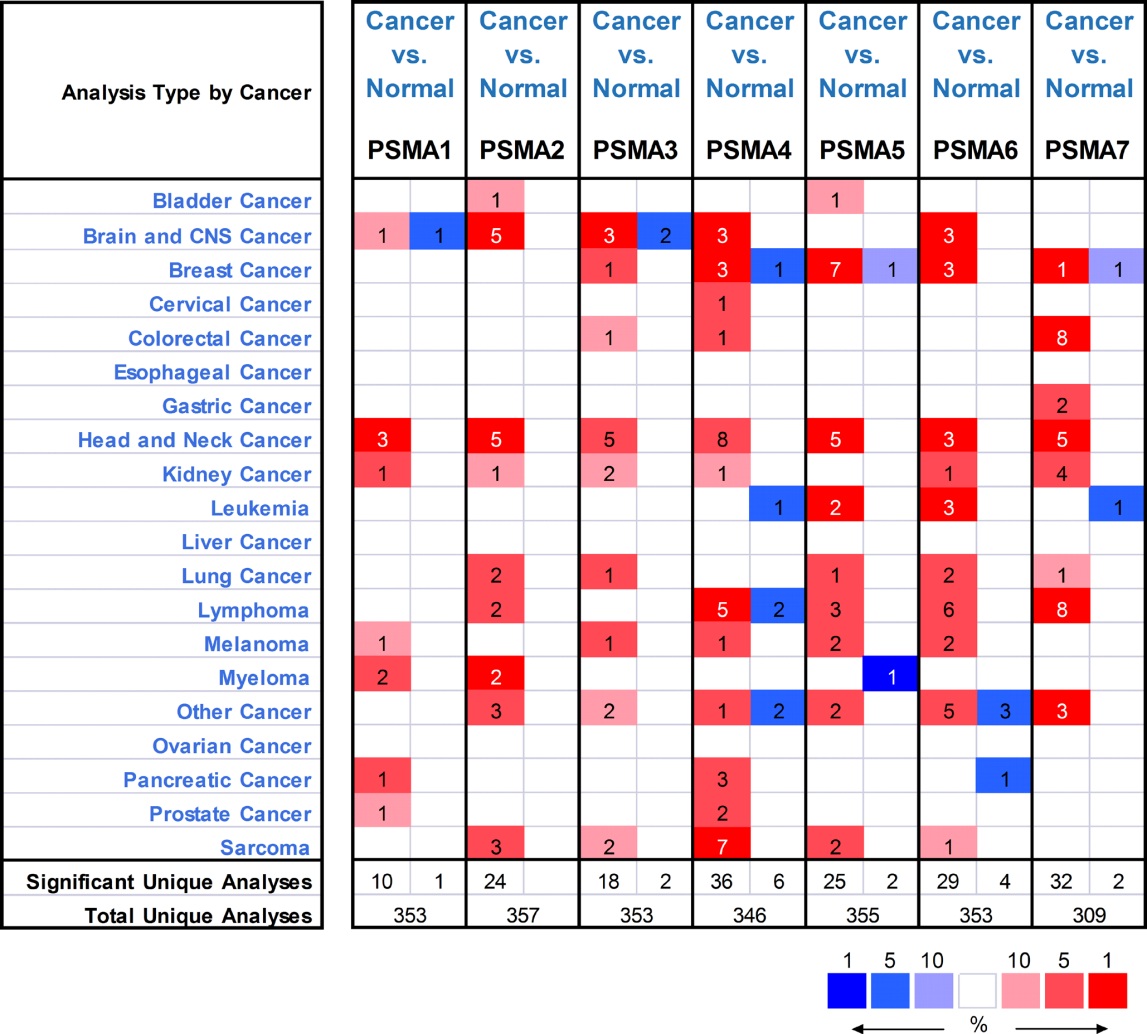
The mRNA expression patterns of PSMAs in overall cancers The mRNA expression difference between tumors and normal tissues were analyzed in Oncomine database with thresholds as follows: *p-*value: 0.01; fold change: 2; gene rank: 10%; data type: mRNA. The number in the colored cell represents the number of analyses meeting these thresholds. The color depth was determined by the gene rank. The red cells indicate that the mRNA levels of target genes are higher in tumor tissues than in normal tissues, while blue cells indicate that the mRNA levels of target genes are lower in tumor tissues than in normal tissues.

Oncomine was used to investigate the mRNA expression differences of the seven PSMAs between tumor and normal tissues in multiple cancers. As shown in Figure 1, the database contained a total of 353, 357, 353, 346, 355, 353 and 309 unique analyses for PSMA1, PSMA2, PSMA3, PSMA4, PSMA5, PSMA6 and PSMA7, respectively. There were 11 studies showing a significant statistical difference for PSMA1, of which 10 showed that mRNA expression level of PSMA1 was increased in tumor than normal tissues in seven kinds of cancers, while one regarding brain and CNS cancer showed an opposite result. As for PSMA2, all 24 datasets with statistical significance revealed higher expression levels of PSMA2 in cancer tissues than in normal tissues. 18 analyses showed increased expression of PSMA3 in tumors, while two showed a significantly decreased expression level in brain and CNS cancer. Compared with normal tissues, PSMA4 was expressed at a much higher level in tumors, demonstrated by 36 analyses involving nine kinds of carcinomas, but six studies showed a reduced expression level of PSMA4 in breast cancer, leukemia, lymphoma and two other cancers. PSMA5 was shown to be upregulated in nine types of cancers by 25 studies and downregulated in breast cancer and myeloma by two analyses. Overexpression of PSMA6 and PSMA7 was found in tumors compared with normal tissues based on 29 and 32 studies, respectively. Meanwhile, only four studies for PSMA6 and two for PSMA7 showed that the mRNA expression level in tumors was lower than in normal samples. Together, among all the datasets with significantly statistical differences, most revealed higher transcription levels of the seven genes in tumors than in normal tissues.

### Transcription levels and prognostic significance of PSMAs in breast cancer

**Figure 2:**
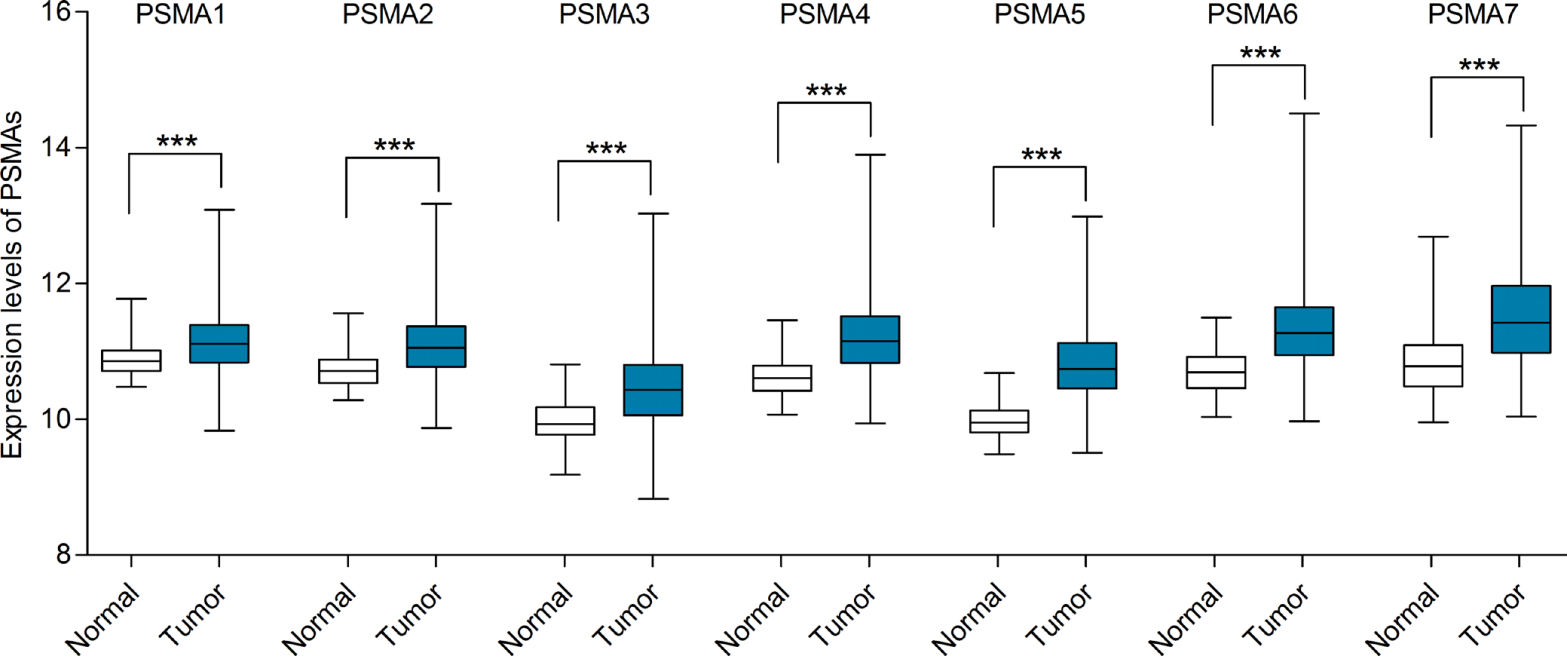
mRNA expression levels of PSMAs in breast cancer (TCGA mRNA HiSeq expression data) mRNA expression levels of PSMAes were investigated in 1095 breast cancer tissues and 113 normal tissues. The line in the middle represents the median value. Statistical differences were examined by two tailed Student's *t*-test. ***p < 0.001.

We first analyzed the mRNA expression level of PSMAs in breast cancer in Oncomine database via cancer vs. normal analysis. There were a total of 13 datasets comparing the mRNA expression level differences between tumors and normal tissues in breast cancer. Among the 13 datasets, all of them were available for PSMA1-3 and PSMA5-6, while only 10 datasets for PSMA4 and nine for PSMA7. For both PSMA1 and PSMA2, no datasets revealed significant difference between the breast cancer group and normal tissue group (Figure 1). PSMA3 was found to be significantly elevated in invasive lobular breast carcinoma compared with normal tissues in Radvanyi's dataset [20]. PSMA4 was upregulated in invasive ductal breast carcinoma and lobular breast carcinoma in datasets from Zhao [21] and Radvanyi [20], while it was downregulated in invasive breast cancer compared with normal breast tissues in Finak's dataset [22]. In a group of datasets including Perou [23], Curtis [24], Sorlie [25], Sorlie 2 [26], Radvanyi, and Zhao [21], the mRNA level of PSMA5 was significantly overexpressed in cancer tissues. However, Finak's dataset [22] showed an opposite result for PSMA5. According to Perou's dataset [23] and two analyses of Sorlie [25], PSMA6 was markedly elevated in ductal breast carcinoma compared to normal tissues. The mRNA level of PSMA7 was higher in breast cancer than in normal samples in Richardson's datasets 2 [27], but was lower in Finak's study [22]. All of the results with statistical significance are summarized in Table 1. Then, the mRNA HiSeq expression data of TCGA was utilized to further determine the expression of the seven PSMAs in breast cancer. As shown in Figure 2, all of the seven genes were significantly overexpressed in 1095 cases of breast cancer compared with 113 normal samples.

**Table 1:** Analyses of PSMAs in breast cancer

Gene	Dataset	Normal (Cases)	Tumor (Cases)	Fold change	*t*-Test	*p*-value
PSMA3	Radvanyi Breast	Breast (6)	Invasive Lobular Breast Carcinoma (5)	2.722	3.308	7.00E-03
PSMA4	Zhao Breast	Breast (3)	Invasive Ductal Breast Carcinoma(38)	2.326	10.982	6.36E-13
		Breast (3)	Lobular Breast Carcinoma (19)	2.283	8.226	3.87E-08
	Radvanyi Breast	Breast (8)	Invasive Ductal Breast Carcinoma(30)	2.317	3.597	3.00E-03
	Finak Breast	Breast (6)	Invasive Breast Carcinoma (53)	−15.502	−22.062	2.74E-29
PSMA5	Perou Breast	Breast (3)	Ductal Breast Carcinoma(36)	2.237	14.385	3.58E-16
	Curtis Breast	Breast (114)	Ductal Breast Carcinoma *in Situ* (10)	2.023	7.295	1.72E-05
		Breast (114)	Medullary Breast Carcinoma (32)	2.016	9.981	5.41E-12
	Sorlie Breast	Breast (4)	Ductal Breast Carcinoma(65)	2.031	6.165	1.00E-03
	Sorlie Breast 2	Breast (4)	Ductal Breast Carcinoma(89)	2.051	7.514	8.58E-04
	Radvanyi Breast	Breast (9)	Invasive Mixed Breast Carcinoma (3)	2.174	2.879	9.00E-03
	Zhao Breast	Breast (3)	Lobular Breast Carcinoma (21)	2.454	8.240	2.23E-04
	Finak Breast	Breast (6)	Invasive Breast Carcinoma (53)	−6.410	−17.907	7.88E-20
PSMA6	Perou Breast	Breast (3)	Ductal Breast Carcinoma(36)	2.287	13.654	4.65E-16
	Sorlie Breast	Breast (4)	Ductal Breast Carcinoma(65)	2.094	7.702	2.55E-04
	Sorlie Breast 2	Breast (4)	Ductal Breast Carcinoma(92)	2.070	8.936	2.38E-04
PSMA7	Richardson Breast 2	Breast (7)	Ductal Breast Carcinoma(40)	2.585	11.321	1.61E-12
	Finak Breast	Breast (6)	Invasive Breast Carcinoma (53)	−13.894	−17.859	8.57E-21

Subsequently, the prognostic effects of PSMAs were determined in KM Plotter database (www.kmplot.com). The desired probe IDs for each gene are shown in Supplementary Table S1. The results showed that high expression of PSMA1 (HR = 1.48; 95% CI: 1.32–1.67; *p* < 0.001), PSMA2 ( HR = 1.14; 95% CI: 1.02–1.28; *p* = 0.021), PSMA3 (HR = 1.34, 95% CI: 1.19–1.50; *p* < 0.001), PSMA4 (HR = 1.53; 95% CI: 1.36–1.71; *p <* 0.001), PSMA5 (HR = 0.71; 95% CI: 0.60–0.84; *p <* 0.001), PSMA6 (HR = 1.39; 95% CI: 1.24–1.56; *p <* 0.001) and PSMA7 (HR = 1.50; 95% CI: 1.33–1.68; *p <* 0.001) were all significantly associated with relapse free survival (RFS) (Table 2). Increased mRNA levels of PSMA5 and PSMA7 were also related to overall survival (OS) with HR = 0.58 (0.41–0.83), *p* = 0.003 and HR = 1.52 (1.20–1.93), *p* < 0.001, respectively, and distant metastasis free survival (DMFS) with HR = 0.61 (0.44-0.85), *p* = 0.003 and HR = 1.32 (1.08-1.62), *p* = 0.007, respectively, but not post progression survival (PPS) (Table 2). In recent years, four intrinsic biological subtypes of breast cancer including luminal A, luminal B, HER2-enriched and basal-like have been revealed by comprehensive transcriptional profiling studies and have been shown to be robust for predicting treatment sensitivity and survival outcomes [23, 28]. Therefore, subgroup analyses based on these four intrinsic subtypes were carried out. Intriguingly, upregulated PSMA1-4 and PSMA6-7 were all significantly associated with worse RFS in the luminal A and B groups, but not in the basal-like or HER2-enriched group. In addition, high expression of PSMA5 was associated with better prognosis in patients with the luminal A or basal-like types, which was consistent with the overall cohort. The results of the subgroup analyses are summarized in Supplementary Table S2.

**Table 2:** Correlation of PSMAs with survival outcomes in breast cancer patients

Gene	Affymetrix ID	Survival outcome	No. of cases	Cut-off value	HR	95% CI	*p*-value
PSMA1	211746_x_at	OS	1117	4585	1.18	0.94–1.50	0.160
		RFS	3554	4803	1.48	1.32–1.67	**< 0.001**
		DMFS	1609	5073	1.21	0.99–1.48	0.067
		PPS	351	4606	0.97	0.75–1.26	0.830
PSMA2	201316_at	OS	1117	630	0.86	0.68–1.09	0.202
		RFS	3554	609	1.14	1.02–1.28	**0.021**
		DMFS	1609	669	0.89	0.73–1.09	0.275
		PPS	351	616	0.85	0.66–1.11	0.232
PSMA3	201532_at	OS	1117	2981	1.02	0.81–1.29	0.858
		RFS	3554	2893	1.34	1.19–1.50	**< 0.001**
		DMFS	1609	3178	0.92	0.75–1.12	0.390
		PPS	351	2879	1.15	0.89–1.49	0.294
PSMA4	203396_at	OS	1117	3423	1.22	0.96–1.55	0.099
		RFS	3554	3069	1.53	1.36–1.71	**< 0.001**
		DMFS	1609	3343	1.19	0.97–1.45	0.095
		PPS	351	3440	1.12	0.87–1.45	0.372
PSMA5	230300_at	OS	522	241	0.58	0.41–0.83	**0.003**
		RFS	1660	215	0.71	0.60–0.84	**< 0.001**
		DMFS	664	233	0.61	0.44–0.85	**0.003**
		PPS	140	225	0.89	0.61–1.31	0.560
PSMA6	208805_at	OS	1117	7028	1.01	0.80–1.28	0.939
		RFS	3554	6687	1.39	1.24–1.56	**< 0.001**
		DMFS	1609	7851	0.97	0.79–1.18	0.740
		PPS	351	7157	0.82	0.63–1.06	0.124
PSMA7	201114_x_at	OS	1117	4160	1.52	1.20–1.93	**< 0.001**
		RFS	3554	4070	1.50	1.33–1.68	**< 0.001**
		DMFS	1609	4401	1.32	1.08–1.62	**0.007**
		PPS	351	4349	1.02	0.79–1.32	0.885

HR: hazard ratio; CI: confidence interval; OS: overall survival; RFS: relapse free survival; DMFS: distant metastasis free survival; PPS: post progression survival.

### Transcription levels and prognostic significance of PSMAs in lung cancer

**Figure 3:**
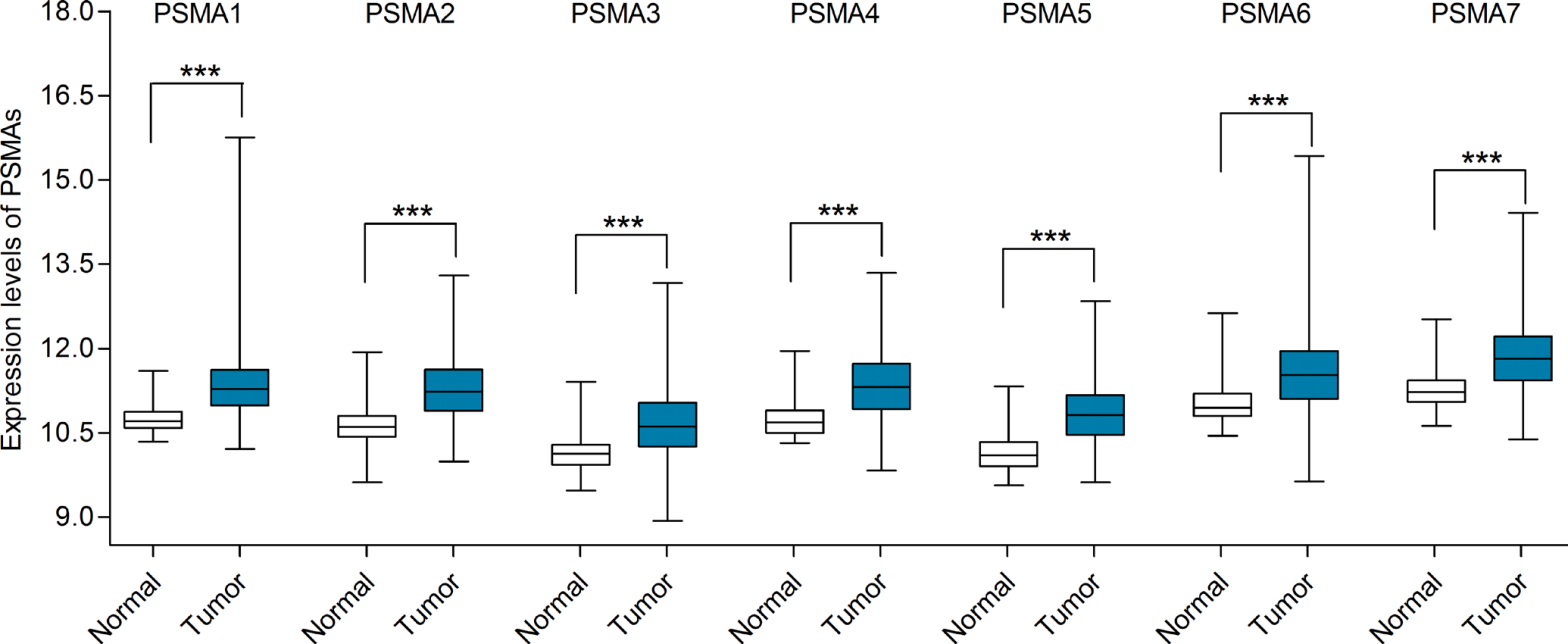
mRNA expression levels of PSMAs in lung cancer (TCGA mRNA HiSeq expression data) mRNA expression levels of PSMAs were investigated in 1013 lung cancer tissues and 109 normal tissues. The line in the middle represents the median value. Statistical differences were examined by two tailed Student's *t*-test. ***p < 0.001.

Likewise, Oncomine database was utilized to compare the mRNA expression levels of PSMAs in lung cancer and normal tissues. With our thresholds (*p*-value = 0.01; fold change = 2; gene rank: 10%, data type: mRNA), none of the datasets revealed statistically significant differences between lung cancer group and normal tissue group for PSMA1 or PSMA4. Two comparisons of Bhattacharjee's dataset [29] indicated that the PSMA2 mRNA levels were higher in small cell lung cancer and lung carcinoid tumor tissues than in normal samples. In Yamagata's dataset [30] analyzing large cell lung carcinoma vs. normal tissue, the mRNA expression level of PSMA3 was significantly upregulated in tumor tissues. PSMA5 was also shown to be overexpressed in lung cancer according to Garber's datasets [31]. Two studies of Yamagata [30] showed that the expression level of PSMA6 was significantly elevated in lung adenocarcinoma and large cell lung carcinoma compared to normal lung tissues. As for PSMA7, the mRNA expression level was dramatically elevated in squamous cell lung carcinoma [29]. All of the results with statistically significant results are shown in Table 3. However, we noticed that the sample sizes of these datasets were relatively small. For instance, there were only five tumor cases against three normal controls in Yamagata's dataset [30]. This may diminish the statistical differences between tumor and normal tissues. Therefore, we further examined the expression differences between lung cancer and normal tissues with TCGA mRNA HiSeq expression data. There were 109 normal samples and 1013 lung cancer samples, including 511 lung adenocarcinomas and 502 lung squamous cell carcinomas. As shown in Figure 3, the expression of all seven PSMAs in lung cancer tissues was remarkably higher than in normal tissues. Next, the mRNA expression levels of PSMAs in normal tissues were separately compared with lung adenocarcinoma and lung squamous cell carcinoma. In line with the overall comparison, the transcription levels of PSMAs were significantly increased in both lung adenocarcinoma and lung squamous cell carcinoma (Supplementary Figure S1).

**Table 3:** Analyses of PSMAs in lung cancer

Gene	Dataset	Normal (cases)	Tumor (cases)	Fold change	*t*-Test	*p*-value
PSMA2	Bhattacharjee Lung	Lung (17)	Small Cell Lung Carcinoma (6)	2.435	3.409	2.00E-03
		Lung (17)	Lung Carcinoid Tumor (20)	2.321	3.242	2.00E-03
PSMA3	Yamagata Lung	Lung (3)	Large Cell Lung Carcinoma (5)	2.011	4.233	3.00E-03
PSMA5	Garber Lung	Lung (5)/Fetal Lung (1)	Large Cell Lung Carcinoma (4)	2.172	3.970	3.00E-03
PSMA6	Yamagata Lung	Lung (3)	Lung Adenocarcinoma (8)	2.561	3.218	6.00E-03
		Lung (3)	Large Cell Lung Carcinoma (5)	2.529	3.823	6.00E-03
PSMA7	Bhattacharjee Lung	Lung (17)	Squamous Cell Lung Carcinoma (21)	7.285	2.810	4.00E-03

We then assessed the prognostic values of PSMAs for lung cancer in KM Plotter database. OS, first progression (FP) and post progression survival (PPS) were analyzed for each gene. High mRNA expression of PSMA1 and PSMA2 was significantly associated with PPS for lung cancer patients, with HR = 0.77 (0.60–0.99), *p* = 0.043 and HR = 0.65 (0.51–0.84), *p* < 0.001, respectively. PSMA3 was found to be uncorrelated with OS, FP or PPS for lung cancer patients. In addition, high mRNA level of PSMA4 was significantly associated with FP (HR = 0.80; 95% CI: 0.66–0.97; *p* = 0.021) but not OS or PPS. Interestingly, increased mRNA level of PSMA5 predicted better OS (HR = 0.51; 95% CI: 0.43–0.60; *p* < 0.001), FP (HR = 0.71; 95% CI: 0.54–0.93; *p* = 0.012) and PPS (HR = 0.64; 95% CI: 0.42–0.99; *p* = 0.042). On the contrary, high PSMA6 expression was associated with worse OS (HR = 1.33; 95% CI: 0.1.17–1.51; *p* < 0.001) and FP (HR = 1.37; 95% CI: 1.13–1.66; *p* = 0.001) but not PPS for lung cancer patients. PSMA7 was also found to be significantly associated with worse OS (HR = 1.28; 95% CI: 1.13–1.45; *p* < 0.001) but not FS or PPS. The prognostic effects of the seven genes are summarized in Table 4. In a further analysis, patients were stratified by histological type, tumor stage and tumor grade (Supplementary Table S3). The results showed that most of the PSMAs were significantly correlated with prognosis in adenocarcinoma, but not in squamous cell carcinoma. When grouped by tumor stage, all seven PSMAs showed different prognostic values in stage 1, whereas only PSMA1, PSMA2 and PSMA5 were correlated with OS or PPS in stage 2. Finally, analyses were performed for tumor grades I and II. However, only PSMA1 and PSMA3 were significantly associated with poor outcomes in patients with tumor grade II.

**Table 4:** Correlation of PSMAs with survival outcomes in lung cancer patients

Gene	Affymetrix ID	Survival outcome	No. of cases	Cut-off value	HR	95% CI	*p*-value
PSMA1	211746_x_at	OS	1926	5033	0.94	0.83–1.06	0.319
		FP	982	5033	0.83	0.69–1.00	0.055
		PPS	344	4645	0.77	0.60–0.99	**0.043**
PSMA2	201316_at	OS	1926	547	0.89	0.79–1.01	0.075
		FP	982	474	0.91	0.76–1.11	0.361
		PPS	344	437	0.65	0.51–0.84	**< 0.001**
PSMA3	201532_at	OS	1926	3495	1.08	0.95–1.23	0.213
		FP	982	2988	1.12	0.93–1.36	0.227
		PPS	344	2871	0.83	0.64–1.07	0.142
PSMA4	203396_at	OS	1926	3381	0.99	0.88–1.13	0.930
		FP	982	2943	0.80	0.66–0.97	**0.021**
		PPS	344	2460	1.13	0.87–1.45	0.359
PSMA5	230300_at	OS	1145	138	0.51	0.43–0.60	**< 0.001**
		FP	596	212	0.71	0.54–0.93	**0.012**
		PPS	138	313	0.64	0.42–0.99	**0.042**
PSMA6	208805_at	OS	1926	7466	1.33	1.17–1.51	**< 0.001**
		FP	982	7080	1.37	1.13–1.66	**0.001**
		PPS	344	7095	1.21	0.94–1.56	0.132
PSMA7	201114_x_at	OS	1926	4431	1.28	1.13–1.45	**< 0.001**
		FP	982	3799	0.89	0.74–1.08	0.235
		PPS	344	3737	1.20	0.93–1.55	0.151

HR: hazard ratio; CI: confidence interval; OS: overall survival; FP: first progression; PPS: post progression survival.

### Transcription levels and prognostic significance of PSMAs in gastric cancer

**Figure 4:**
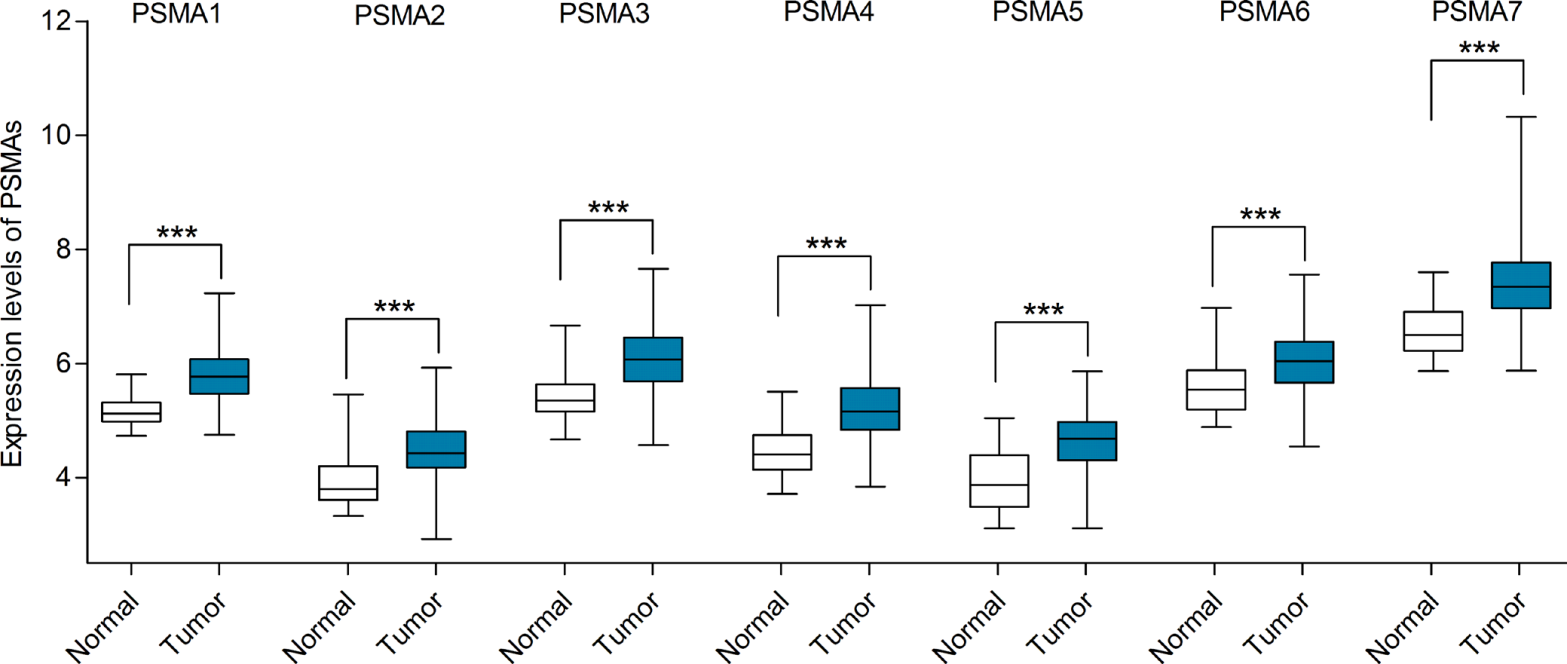
mRNA expression levels of PSMAs in gastric cancer (TCGA mRNA HiSeq expression data) mRNA expression levels of PSMAs were investigated in 384 gastric cancer tissues and 37 normal tissues. The line in the middle represents the median value. Statistical differences were examined by two tailed student's *t*-test. ***p < 0.001.

In Oncomine database, there were five datasets that compared the expression differences between gastric cancer and normal tissues for PSMA1-6, and four datasets for PSMA7. No statistically significant differences of mRNA expression were observed between tumor and normal tissues for PSMA1-6 in any of the five datasets with the thresholds we applied. However, compared with 31 gastric mucosa samples, the mRNA expression level of PSMA7 was significantly upregulated in gastric intestinal type adenocarcinoma (cases = 26, fold change = 2.516, *p* < 0.001) and gastric mixed adenocarcinoma (cases = 4, fold change = 2.555, *p* < 0.001) in DErrico's dataset [32]. Considering the limited number of cases in Oncomine, the TCGA database involving 384 gastric cancer and 37 normal samples were further used to confirm the potential expression difference of PSMAs between tumors and normal tissues. All of the seven genes were remarkably overexpressed in gastric cancer compared with normal tissues (Figure 4).

The correlations between PSMAs and survival outcomes of gastric cancer patients involving OS and FP were then determined in KM Plotter database. The prognostic effects of the seven genes are shown in Table 5. Intriguingly, high mRNA expression levels of all PSMAs were significantly related to better OS with HR = 0.67 (0.56–0.79), *p* <0.001 for PSMA1; HR = 0.65 (0.55–0.78), *p* < 0.001 for PSMA2; HR = 0.77 (0.65–0.91), *p* = 0.002 for PSMA3; HR = 0.66 (0.56–0.79), *p* < 0.001 for PSMA4; HR = 0.80 (0.65–1.00), *p* = 0.047 for PSMA5; HR = 0.62 (0.52–0.73), *p* < 0.001 for PSMA6; HR = 0.64 (0.54–0.76), *p* < 0.001 for PSMA7, and better FP with HR = 0.62 (0.39–0.61), *p* < 0.001 for PSMA1; HR = 0.61 (0.49–0.74), *p* < 0.001 for PSMA2; HR = 0.69 (0.57–0.85), *p* < 0.001 for PSMA3; HR = 0.64 (0.52–0.78), *p* < 0.001 for PSMA4; HR = 0.66 (0.52-0.84), *p* < 0.001 for PSMA5; HR = 0.58 (0.48–0.72), *p* < 0.001 for PSMA6; and HR = 0.70 (0.57–0.85), *p* < 0.001 for PSMA7. Next, the prognostic ability of PSMAs expression was investigated in different tumor stages and HER2 status of gastric cancer. As shown in Supplementary Table S4, a high expression of PSMA1-4 and PSMA6-7 predicted better prognosis in stage 3, but only PSMA6, PSMA5 and PSMA7 were associated with outcomes in stage 1, stage 2 and stage 4, respectively. In line with the overall cohort, the expression of all PSMAs was significantly correlated with better prognosis in the HER2-negative group. However, only PSMA2, PSMA5 and PSMA7 expression were correlated with OS or FP in the HER2-positive group.

**Table 5:** Correlation of PSMAs with survival outcomes in gastric cancer patients

Gene	Affymetrix ID	Survival outcome	No. of cases	Cut-off value	HR	95% CI	*p*-value
PSMA1	211746_x_at	OS	876	438	0.67	0.56–0.79	**< 0.001**
		FP	641	321	0.62	0.39–0.61	**< 0.001**
PSMA2	201316_at	OS	876	438	0.65	0.55–0.78	**< 0.001**
		FP	641	321	0.61	0.49–0.74	**< 0.001**
PSMA3	201532_at	OS	876	437	0.77	0.65–0.91	**0.002**
		FP	641	320	0.69	0.57–0.85	**< 0.001**
PSMA4	203396_at	OS	876	438	0.66	0.56–0.79	**< 0.001**
		FP	641	321	0.64	0.52–0.78	**< 0.001**
PSMA5	230300_at	OS	631	315	0.80	0.65–1.00	**0.047**
		FP	522	260	0.66	0.52–0.84	**< 0.001**
PSMA6	208805_at	OS	876	438	0.62	0.52–0.73	**< 0.001**
		FP	641	321	0.58	0.48–0.72	**< 0.001**
PSMA7	201114_x_at	OS	876	438	0.64	0.54–0.76	**< 0.001**
		FP	641	320	0.70	0.57–0.85	**< 0.001**

HR: hazard ratio; CI: confidence interval; OS: overall survival; FP: first progression.

### Transcription levels and prognostic significance of PSMAs in several other cancers

As for ovarian cancer, the Oncomine database revealed no significant differences in the mRNA expression of PSMAs between ovarian tumors and normal tissues (Figure 1). Meanwhile, the mRNA levels of PSMAs could not be compared between tumors and normal tissues in TCGA database due to the lack of normal ovarian samples. Next, we examined the prognostic significance of PSMAs in KM Plotter database, and the results showed that only PSMA1 was associated with PPS (HR = 0.83; 95% CI: 0.70–1.00; *p* = 0.044) for ovarian cancer patients (Table 6).

**Table 6:** Correlation of PSMAs with survival outcomes in ovarian cancer patients

Gene	Affymetrix ID	Survival outcome	No. of cases	Cut-off value	HR	95% CI	*p*-value
PSMA1	211746_x_at	OS	1582	4834	1.01	0.88–1.15	0.893
		PFS	1306	4898	1.03	0.90–1.17	0.660
		PPS	708	5025	0.83	0.70–1.00	**0.044**
PSMA2	201316_at	OS	1582	711	1.12	0.98–1.27	0.109
		PFS	1306	724	0.96	0.84–1.09	0.514
		PPS	708	725	1.03	0.86–1.23	0.759
PSMA3	201532_at	OS	1582	3015	1.02	0.89–1.17	0.758
		PFS	1306	3186	1.13	0.99–1.29	0.072
		PPS	708	3323	1.02	0.85–1.22	0.859
PSMA4	203396_at	OS	1582	3779	0.98	0.85–1.12	0.717
		PFS	1306	3840	0.99	0.87–1.13	0.853
		PPS	708	3946	0.93	0.78–1.12	0.453
PSMA5	230300_at	OS	580	164	0.91	0.73–1.14	0.428
		PFS	484	150	0.85	0.69–1.05	0.125
		PPS	307	146	0.99	0.76–1.30	0.944
PSMA6	208805_at	OS	1582	7678	1.02	0.89–1.17	0.771
		PFS	1306	8209	1.08	0.94–1.23	0.279
		PPS	708	8516	0.9	0.75–1.08	0.251
PSMA7	201114_x_at	OS	1582	4333	1.12	0.98–1.28	0.091
		PFS	1306	4671	1.11	0.97–1.27	0.121
		PPS	708	4793	0.92	0.77–1.10	0.381

HR: hazard ratio; CI: confidence interval; OS: overall survival; PFS: progression free survival; PPS: post progression survival.

**Figure 5:**
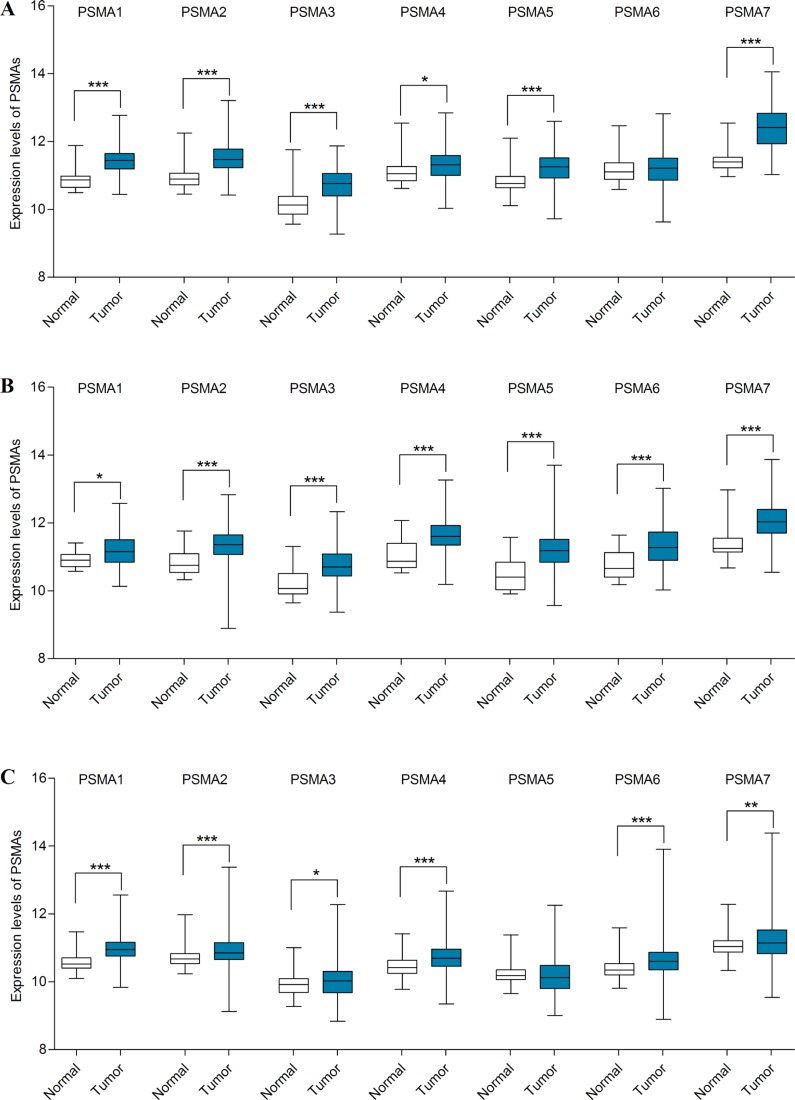
mRNA expression levels of PSMAs in colorectal, bladder and kidney cancer (TCGA mRNA HiSeq expression data) (**A**) mRNA expression levels of PSMAs were investigated in 380 colorectal cancers and 50 normal tissues. (**B**) mRNA expression levels of PSMAs were investigated in 407 bladder cancers and 19 normal tissues. (**C**) mRNA expression levels of PSMAs were investigated in 889 kidney cancers and 129 normal tissues. The line in the middle represents the median value. Statistical differences were examined by two tailed Student's *t*-test. *p < 0.05; **p < 0.01; ***p < 0.001.

In colorectal cancer, it was previously reported that the mRNA and protein expression levels of PSMA7 were much higher than in normal tissue, and a high protein expression of PSMA7 was significantly associated with worse OS for colorectal cancer patients [19]. In line with this study, eight comparisons from five datasets including Ki [33], Skrzypczak [34], TCGA, Skrzypczak 2 [34] and Hong [35] revealed that the mRNA level of PSMA7 was increased in different types of colorectal cancer compared with normal tissues. Moreover, PSMA3 and PSMA4 were also upregulated in rectal adenoma compared with normal tissues from Sabates-Bellver's datasets [36] (Table 7). In the TCGA database, PSMA1-5 and PSMA7 were overexpressed in colorectal cancer tissues examined by mRNA HiSeq expression data (Figure 5A). Alternatively, the prognostic values of PSMAs were then determined by the PrognScan database because survival data of colorectal cancer was absent in KM Plotter. The probe IDs selected in PrognScan database were consistent with the desired Affymetrix IDs in KM plotter. The results showed that only elevated mRNA of PSMA7 predicted worse DFS (HR = 3.02, 95% CI: 1.34–6.81, *p* = 0.008) in Smith's study [37]. The correlations between the mRNA expression of PSMAs and survival outcomes of colorectal cancer patients reported by PrognScan database are summarized in Table 8.

**Table 7:** Analyses of PMSAs in multiple cancers

Cancer	Gene	Dataset	Normal (Cases)	Tumor (Cases)	Fold change	*t*-Test	*p*-value
Colorectal cancer	PSMA3	Sabates-Bellver Colon	Colon (25)/Rectum (7)	Rectal Adenoma (7)	2.112	7.162	2.47E-05
	PSMA4	Sabates-Bellver Colon	Colon (25)/Rectum (7)	Rectal Adenoma (7)	2.225	9.131	5.40E-06
	PSMA7	Ki Colon	Colon (28)/Liver (13)	Colon Adenocarcinoma (50)	2.124	10.045	2.01E-15
		Skrzypczak Colorectal	Colorectal Tissue (24)	Colorectal Carcinoma (36)	2.111	8.780	1.95E-12
		TCGA Colorectal	Colon (19)/Rectum (3)	Rectal Adenocarcinoma (60)	2.407	13.228	2.88E-20
			Colon (19)/Rectum (3)	Colon Adenocarcinoma (101)	2.463	15.110	2.93E-21
			Colon (19)/Rectum (3)	Cecum Adenocarcinoma (22)	2.044	7.710	3.76E-09
		Skrzypczak Colorectal 2	Colon (10)	Colon Carcinoma (5)	3.018	13.959	1.70E-09
			Colon (10)	Colon Carcinoma (5)	3.125	11.038	1.81E-06
		Hong Colorectal	Colon (12)	Colorectal Carcinoma (70)	3.290	13.308	1.05E-12
Bladder cancer	PSMA2	Dyrskjot Bladder 3	Bladder (9)/Bladder Mucosa (5)	Infiltrating Bladder Urothelial Carcinoma (13)	2.984	5.672	3.12E-05
	PSMA5	Sanchez-Carbayo Bladder 2	Bladder (48)	Superficial Bladder Cancer (28)	2.385	8.739	4.05E-12
Kidney cancer	PSMA1	Jones Renal	Kidney (23)	Renal Pelvis Urothelial Carcinoma (8)	2.128	10.933	5.73E-09
	PSMA2	Jones Renal	Kidney (23)	Renal Pelvis Urothelial Carcinoma (8)	2.325	9.303	8.95E-07
	PSMA3	Jones Renal	Kidney (23)	Renal Pelvis Urothelial Carcinoma (8)	2.198	7.719	2.26E-06
		Yusenko Renal	Fetal Kidney (2)/Kidney (3)	Renal Wilms Tumor (4)	2.144	3.749	4.00E-03
	PSMA4	Jones Renal	Kidney (23)	Renal Pelvis Urothelial Carcinoma (8)	2.179	7.243	1.52E-05
	PSMA6	Yusenko Renal	Fetal Kidney (2)/Kidney (3)	Renal Wilms Tumor (4)	2.192	3.962	3.00E-03
	PSMA7	Higgins Renal	Kidney (2)	Papillary Renal Cell Carcinoma (4)	2.007	4.515	6.00E-03
		Yusenko Renal	Fetal Kidney (2)/Kidney (3)	Chromophobe Renal Cell Carcinoma (4)	3.061	6.192	2.68E-04
			Fetal Kidney (2)/Kidney (3)	Renal Oncocytoma (4)	2.116	5.638	1.00E-03
			Fetal Kidney (2)/Kidney (3)	Renal Wilms Tumor (4)	2.492	3.824	6.00E-03
Melanoma	PSMA1	Haqq Melanoma	Skin (3)	Melanoma (5)	2.229	7.928	6.57E-04
	PSMA3	Haqq Melanoma	Skin (3)	Melanoma (6)	2.235	6.195	3.39E-04
	PSMA4	Talantov Melanoma	Skin (7)	Cutaneous Melanoma (45)	3.418	11.368	1.60E-08
	PSMA5	Haqq Melanoma	Skin (3)	Non-Neoplastic Nevus (9)	2.353	7.140	2.79E-05
			Skin (3)	Melanoma (6)	3.002	5.306	8.61E-04
	PSMA6	Haqq Melanoma	Skin (3)	Non-Neoplastic Nevus (9)	3.005	7.375	7.24E-04
			Skin (3)	Melanoma (6)	3.296	5.624	4.12E-04

**Table 8:** Correlation of PSMAs with survival outcomes in colorectal cancer patients

Gene	Affymetrix ID	Dataset	Survival outcome	No. of cases	HR	95% CI	*p*-value
PSMA1	211746_x_at	GSE12945	OS	62	1.01	0.88−1.15	0.714
			DFS	51	0.80	0.25−2.59	0.145
		GSE17536	OS	177	0.84	0.33−2.16	0.717
			DFS	145	1.14	0.31−4.23	0.849
			DSS	177	0.54	0.19−1.59	0.264
		GSE14333	DFS	226	0.72	0.16−3.12	0.656
		GSE17537	OS	55	2.36	0.57−9.83	0.236
			DFS	55	3.51	0.73−16.86	0.117
			DSS	49	3.21	0.38−27.32	0.285
PSMA2	201316_at	GSE12945	OS	62	0.45	0.11−1.79	0.258
			DFS	51	0.37	0.04−3.14	0.360
		GSE17536	OS	177	1.28	0.66−2.48	0.460
			DFS	145	1.03	0.40−2.67	0.951
			DSS	177	1.09	0.51−2.33	0.831
		GSE14333	DFS	226	0.63	0.30−1.33	0.225
		GSE17537	OS	55	2.74	0.93−8.01	0.066
			DFS	55	2.48	0.77−8.01	0.128
			DSS	49	2.67	0.66−10.76	0.168
PSMA3	201532_at	GSE12945	OS	62	1.23	0.66−2.30	0.522
			DFS	51	0.95	0.41−2.18	0.898
		GSE17536	OS	177	1.72	0.93−3.18	0.083
			DFS	145	1.36	0.59−3.13	0.466
			DSS	177	1.83	0.89−3.73	0.099
		GSE14333	DFS	226	0.91	0.44−1.89	0.798
		GSE17537	OS	55	1.24	0.48−3.22	0.652
			DFS	55	1.16	0.43−3.13	0.766
			DSS	49	1.02	0.25−4.07	0.980
PSMA4	203396_at	GSE12945	OS	62	2.09	0.64−6.78	0.220
			DFS	51	1.37	0.27−7.03	0.706
		GSE17536	OS	177	1.40	0.79−2.48	0.245
			DFS	145	0.79	0.36−1.72	0.547
			DSS	177	1.26	0.65−2.45	0.495
		GSE14333	DFS	226	0.52	0.24−1.13	0.098
		GSE17537	OS	55	1.20	0.35−4.15	0.768
			DFS	55	1.56	0.42−5.88	0.509
			DSS	49	1.77	0.34−9.33	0.500
PSMA5	230300_at	Not available					
PSMA6	208805_at	GSE12945	OS	62	2.38	0.54−10.48	0.253
			DFS	51	2.87	0.30−27.71	0.363
		GSE17536	OS	177	1.40	0.66−2.96	0.380
			DFS	145	0.87	0.31−2.43	0.790
			DSS	177	1.06	0.45−2.48	0.902
		GSE14333	DFS	226	0.42	0.17−1.03	0.059
		GSE17537	OS	55	1.16	0.37−3.66	0.798
			DFS	55	0.98	0.31−3.11	0.976
			DSS	49	1.15	0.19−6.99	0.880
PSMA7	201114_x_at	GSE12945	OS	62	1.32	0.63−2.78	0.466
			DFS	51	1.71	0.52−5.56	0.374
		GSE17536	OS	177	0.85	0.49−1.46	0.555
			DFS	145	0.49	0.22−1.09	0.082
			DSS	177	0.56	0.30−1.06	0.073
		GSE14333	DFS	226	0.61	0.34−1.07	0.086
		GSE17537	OS	55	1.21	0.62−2.35	0.576
			DFS	55	3.02	1.34−6.81	**0.008**
			DSS	49	1.78	0.70−4.50	0.224

HR: hazard ratio; CI: confidence interval; OS: overall survival; DFS: diseases free survival; DSS: disease specific survival.

For bladder cancer, Oncomine included only four datasets that were available for cancer vs. normal analysis, of which Dyrskjot [38] and Sanchez-Carbayo's dataset [39] respectively showed elevated expression levels of PSMA2 and PSMA5 in bladder cancer compared to normal tissues (Table 7). However, all seven genes were overexpressed in cancer tissues examined by mRNA HiSeq expression data in the TCGA database (Figure 5B). In the PrognScan database, only one study for each gene was retrieved, but no significant association was found between PSMAs and OS for bladder cancer patients (Table 9).

**Table 9:** Correlation of PSMAs with survival outcomes in bladder cancer

Gene	Affymetrix ID	Dataset	Survival outcome	No. of cases	HR	95% CI	*p*-value
PSMA1	211746_x_at	GSE5287	OS	30	1.04	0.44−2.46	0.928
PSMA2	201316_at	GSE5287	OS	30	0.28	0.02−3.62	0.329
PSMA3	201532_at	GSE5287	OS	30	1.54	0.51−4.65	0.446
PSMA4	203396_at	GSE5287	OS	30	1.46	0.54−3.96	0.453
PSMA5	Not available						
PSMA6	208805_at	GSE5287	OS	30	1.77	0.59−5.26	0.307
PSMA7	201114_x_at	GSE5287	OS	30	0.9	0.31−2.67	0.854

HR: hazard ratio; CI: confidence interval; OS: overall survival.

In kidney cancer, Jones's dataset [40] showed that the mRNA expression levels of PSMA1-4 were upregulated in renal pelvis urothelial carcinoma compared with normal kidney tissues (Table 7). In Yusenko's dataset [41], the transcription levels of both PSMA3 and PSMA6 were higher in renal Wilms tumor than in normal tissues. PSMA7 was overexpressed in kidney cancer analyzed by Higgins and Yusenko's datasets [41, 42]. Consistent with the trend seen in Oncomine, the expression levels of PSMA1-4 and PSMA6-7 were significantly increased in 889 kidney cancers compared with 129 normal tissues in the TCGA mRNA HiSeq expression data (Figure 5C). Furthermore, analyses were also performed in different subtypes of kidney cancer including chromophobe cell, clear cell and papillary cell carcinoma by the TCGA data (Supplementary Figure S2). Compared with normal kidney tissue, PSMA1, PSMA3 and PSMA7 were upregulated in kidney chromophobe cell carcinoma, whereas PSMA5 was downregulated. The expression levels of PSMA1-3 and PSMA6 were higher in kidney clear cell carcinoma than in normal samples. Except for PSMA3, all PSMAs were significantly upregulated in kidney papillary cell carcinoma compared with normal tissue. However, since survival data was not available in the KM Plotter or PrognScan databases, we were unable to investigate the prognostic effects of PSMAs in kidney cancer.

With respect to melanoma, the mRNA expression levels of PSMA1, PSMA3, PSMA5 and PSMA6 were upregulated in melanoma compared with normal tissues according to Haqq's dataset [43] (Table 7). In Talantov's dataset [44], PSMA4 was higher in 45 cutaneous melanomas than in seven normal skin samples. However, we were not able to compare the expression difference of PSMAs between tumors and normal tissues in the TCGA database due to the lack of normal samples. Subsequently, the PrognScan database was used to investigate the prognostic significance of the PSMAs in melanoma. As shown in Table 10, PSMA1, PSMA3 and PSMA7 were significantly associated with survival outcomes in melanoma patients [45, 46].

**Table 10:** Correlation of PSMAs with survival outcomes in melanoma patients

Gene	Affymetrix ID	Dataset	Cancer type	Survival outcome	No. of cases	HR	95% CI	*p*-value
PSMA1	211746_x_at	GSE22138	Uveal melanoma	DMFS	63	1.58	0.96−2.62	0.074
		GSE19234	Skin melanoma	OS	38	18.53	2.68−128.33	**0.003**
PSMA2	201316_at	GSE22138	Uveal melanoma	DMFS	63	1.14	0.75−1.74	0.526
		GSE19234	Skin melanoma	OS	38	1.99	0.72−5.50	0.182
PSMA3	201532_at	GSE22138	Uveal melanoma	DMFS	63	1.55	1.06−2.28	**0.024**
		GSE19234	Skin melanoma	OS	38	1.10	0.23−5.30	0.907
PSMA4	203396_at	GSE22138	Uveal melanoma	DMFS	63	1.18	0.89−1.55	0.246
		GSE19234	Skin melanoma	OS	38	3.48	0.58−20.81	0.172
PSMA5	230300_at	Not available						
PSMA6	208805_at	GSE22138	Uveal melanoma	DMFS	63	1.53	0.90−2.62	0.119
		GSE19234	Skin melanoma	OS	38	2.04	0.31−13.33	0.459
PSMA7	201114_x_at	GSE22138	Uveal melanoma	DMFS	63	4.53	1.74−11.76	**0.002**
		GSE19234	Skin melanoma	OS	38	9.44	1.77−50.33	**0.009**

HR: hazard ratio; CI: confidence interval; OS: overall survival; DMFS: distant metastasis free survival.

In head and neck cancer, there were a total of 15 datasets investigating PSMAs mRNA expression in tumor and normal tissues in Oncomine database. In Pyeon's multi-cancer datasets [47], the mRNA expression levels of all seven genes were shown to be upregulated in several kinds of head and neck cancers compared with normal tissues (Table 11). PSMA4 mRNA was also found to be significantly elevated in datasets including Frierson [48], Talbot [49], Cromer [50] and Estilo [51]. In addition, Ginos's dataset [52] showed an increased expression level of PSMA5 in head and neck squamous cell carcinoma. However, since the survival data was absent in KM Plotter and PrognScan, we could not investigate the prognostic effects of PSMAs in head and neck cancer.

**Table 11:** Analyses of PMSAs in head and neck cancer

Gene	Dataset	Normal (Cases)	Tumor (Cases)	Fold change	*t*-Test	*p*-value
PSMA1	Pyeon Multi-cancer	Cervix Uteri (8)/Oral Cavity (9)/Palate (1)/Tonsil (4)	Oropharyngeal Carcinoma (6)	2.701	5.992	4.34E-06
		Cervix Uteri (8)/Oral Cavity (9)/Palate (1)/Tonsil (4)	Floor of the Mouth Carcinoma (5)	3.025	5.833	5.00E-05
		Cervix Uteri (8)/Oral Cavity (9)/Palate (1)/Tonsil (4)	Tongue Carcinoma (15)	2.106	4.358	5.61E-05
PSMA2	Pyeon Multi-cancer	Cervix Uteri (8)/Oral Cavity (9)/Palate (1)/Tonsil (4)	Oropharyngeal Carcinoma (6)	3.574	8.266	6.05E-09
		Cervix Uteri (8)/Oral Cavity (9)/Palate (1)/Tonsil (4)	Tongue Carcinoma (15)	3.107	6.079	3.04E-07
		Cervix Uteri (8)/Oral Cavity (9)/Palate (1)/Tonsil (4)	Floor of the Mouth Carcinoma (5)	3.803	7.187	6.12E-07
		Cervix Uteri (8)/Oral Cavity (9)/Palate (1)/Tonsil (4)	Tonsillar Carcinoma (6)	2.227	4.070	3.69E-04
		Cervix Uteri (8)/Oral Cavity (9)/Palate (1)/Tonsil (4)	Oral Cavity Carcinoma (4)	3.049	4.748	9.08E-04
PSMA3	Pyeon Multi-cancer	Cervix Uteri (8)/Oral Cavity (9)/Palate (1)/Tonsil (4)	Oropharyngeal Carcinoma (6)	4.452	5.399	1.41E-05
		Cervix Uteri (8)/Oral Cavity (9)/Palate (1)/Tonsil (4)	Oral Cavity Carcinoma (4)	4.449	5.255	8.79E-05
		Cervix Uteri (8)/Oral Cavity (9)/Palate (1)/Tonsil (4)	Tongue Carcinoma (15)	3.773	5.050	7.08E-06
		Cervix Uteri (8)/Oral Cavity (9)/Palate (1)/Tonsil (4)	Floor of the Mouth Carcinoma (5)	5.384	5.462	6.19E-05
		Cervix Uteri (8)/Oral Cavity (9)/Palate (1)/Tonsil (4)	Tonsillar Carcinoma (6)	2.060	2.685	7.00E-03
PSMA4	FriersonHF Salivary-gland	Salivary Gland (6)	Salivary Gland Adenoid Cystic Carcinoma (16)	2.887	6.997	7.82E-07
	Pyeon Multi-cancer	Cervix Uteri (8)/Oral Cavity (9)/ Palate (1)/Tonsil (4)	Oropharyngeal Carcinoma (6)	3.181	6.897	1.17E-05
		Cervix Uteri (8)/Oral Cavity (9)/Palate (1)/Tonsil (4)	Oral Cavity Carcinoma (4)	2.549	6.087	1.22E-04
		Cervix Uteri (8)/Oral Cavity (9)/Palate (1)/Tonsil (4)	Tongue Carcinoma (15)	2.225	5.311	4.13E-06
		Cervix Uteri (8)/Oral Cavity (9)/Palate (1)/Tonsil (4)	Floor of the Mouth Carcinoma (5)	2.208	5.473	5.89E-05
	Talbot Lung	Lung (2)/Tongue (26)	Tongue Squamous Cell Carcinoma (31)	2.120	7.290	1.38E-09
	Cromer Head-Neck	Uvula (4)	Head and Neck Squamous Cell Carcinoma (34)	2.059	4.616	7.90E-04
	Estilo Head-Neck	Tongue (26)	Tongue Squamous Cell Carcinoma (31)	2.107	6.810	1.42E-08
PSMA5	Pyeon Multi-cancer	Cervix Uteri (8)/Oral Cavity (9)/Palate (1)/Tonsil (4)	Oropharyngeal Carcinoma (6)	3.032	7.548	2.44E-07
		Cervix Uteri (8)/Oral Cavity (9)/Palate (1)/Tonsil (4)	Tongue Carcinoma (15)	2.523	5.569	2.03E-06
		Cervix Uteri (8)/Oral Cavity (9)/Palate (1)/Tonsil (4)	Floor of the Mouth Carcinoma (5)	2.697	6.255	1.72E-05
		Cervix Uteri (8)/Oral Cavity (9)/Palate (1)/Tonsil (4)	Oral Cavity Carcinoma (4)	2.540	5.068	7.64E-04
	Ginos Head-Neck	Buccal Mucosa (13)	Head and Neck Squamous Cell Carcinoma (41)	2.195	11.995	5.42E-15
PSMA6	Pyeon Multi-cancer	Cervix Uteri (8)/Oral Cavity (9)/Palate (1)/Tonsil (4)	Tongue Carcinoma (15)	2.275	6.267	1.85E-07
		Cervix Uteri (8)/Oral Cavity (9)/Palate (1)/Tonsil (4)	Oral Cavity Carcinoma (4)	2.388	5.998	1.15E-04
		Cervix Uteri (8)/Oral Cavity (9)/Palate (1)/Tonsil (4)	Oropharyngeal Carcinoma (6)	2.503	5.214	2.16E-04
PSMA7	Pyeon Multi-cancer	Cervix Uteri (8)/Oral Cavity (9)/Palate (1)/Tonsil (4)	Tongue Carcinoma (15)	2.660	7.577	3.60E-09
		Cervix Uteri (8)/Oral Cavity (9)/Palate (1)/Tonsil (4)	Oral Cavity Carcinoma (4)	2.937	8.314	2.14E-07
		Cervix Uteri (8)/Oral Cavity (9)/Palate (1)/Tonsil (4)	Tonsillar Carcinoma (6)	2.028	5.090	3.74E-05
		Cervix Uteri (8)/Oral Cavity (9)/Palate (1)/Tonsil (4)	Oropharyngeal Carcinoma (6)	2.335	5.610	2.67E-05
		Cervix Uteri (8)/Oral Cavity (9)/Palate (1)/Tonsil (4)	Floor of the Mouth Carcinoma (5)	2.540	6.119	2.69E-05

## DISCUSSION

The proteasome is engaged in the degradation of numerous proteins involved in critical physiological functions in human cancers. Elevated proteasome activity has frequently been detected in different types of tumor cells, which is consistent with the fact that malignant cells are generally more sensitive to proteasome inhibitors than non-cancerous cells [53, 54]. Along with high proteasome activity, the expression of many proteasome subunits has been reported to be upregulated in different kinds of tumors compared with normal tissues, thus indicating that elevated proteasome subunits might be the underlying mechanism of high proteasome activity and therefore may represent key points for drugs targeting proteasome in cancer [54–56]. In this context, Deng and colleagues demonstrated that the expression of six proteasome subunits including PSMA1, PSMB5, PSMD1, PSMD2, PSMD8 and PSMD11 was increased over three-fold in breast cancer tissues when compared to adjacent normal tissues [8]. Upregulation of PSMA6, PSMB4, PSMC2 and PSMD12 was also observed in hepatocellular carcinomas in p21-HBx transgenic mice [57]. Combined with higher proteasome activity, increased levels of PSMA5 and PSMAD4 could be detected in colorectal cancer [55]. In this study, we systematically analyzed the mRNA expression levels of PSMA1-7 in multiple human cancers based on Oncomine and TCGA databases. The results showed that the mRNA expression levels of all seven PSMAs were significantly upregulated in breast, lung, gastric, bladder and head and neck cancer compared with normal tissues. In colorectal cancer, PSMA1-5 and PSMA7 were demonstrated to be overexpressed in tumor tissues. Notably, although PSMA1-4 and PSMA6-7 were increased in overall kidney cancer, the transcriptional patterns of PSMAs were different among the three subtypes. For instance, the mRNA expression level of PSMA3 was elevated in kidney chromophobe cell carcinoma, but not in clear cell or papillary cell carcinoma. Moreover, we also revealed that melanoma had higher levels of PSMA1 and PSMA3-6 compared with normal tissues.

It has been reported that many proteasome subunits are significantly associated with various clinicopathological features and survival outcomes for cancer patients. In ovarian cancer, high expression of PSMB4 was closely related to tumor grade, tumor stage, lymph node, ascites and Ki-67, as well as worse OS [11]. Hepatocellular cancer patients with higher expression of PSMD10 were found to be characterized by increased tumor size, vascular invasion as well as intrahepatic or distant metastasis and would suffer a poor OS or shorter DFS than low-expression patients [9]. Langlands *et al*. demonstrated that positive expression of PSMD9 was significantly correlated with higher rates of local recurrence after radiotherapy in breast cancer [58]. Another study reported that overexpression of PSMD2 predicted a poor prognosis for lung adenocarcinoma patients [7].

Nevertheless, as for the PSMAs, only a limited number of studies have investigated the prognostic significance in human cancers. One clue provided by a previous study showed that overexpression of PSMA7 in protein level was significantly associated with liver metastasis and worse prognosis in colorectal cancer [10]. Hence, it is reasonable to speculate that the mRNA expression of certain PSMAs might correlate with survival outcomes for cancer patients. In the present study, we found that high expression of PSMA1-4 and PSMA6-7 was significantly associated with worse prognosis in breast cancer, while PSMA5 was related to better OS, RFS and DMFS. Moreover, we also observed that these significant correlations were specifically present in the luminal A and B subtypes of breast cancer. In lung cancer, PSMA1-2, PSMA4 and PSMA5 were correlated with better prognosis, whereas PSMA6 and PSMA7 predicted worse survival outcomes. However, such correlations might only be applicable to lung adenocarcinoma but not squamous cell carcinoma. Intriguingly, all seven genes were significantly associated with better prognosis for overall gastric cancer and HER2-negative gastric cancer. In ovarian cancer, only PSMA1 was marginally correlated with PPS. Although significant association between certain PSMAs and clinical outcomes was observed in colorectal cancer and melanoma, caution should be taken due to the limited number of cases. Regretfully, survival data for bladder, kidney and head and neck cancer were not available in the KM Plotter or PrognScan databases.

As important components of the proteasome, accumulating evidence indicates that proteasome subunits could exert different biological functions in a proteasome-dependent or independent manner. For example, the most well studied subunit of proteasome, PSMD10, not only performs a proteolytic role in the degradation of multiple proteins, but also plays a non-proteolytic role in transcriptional regulation, protein trafficking and signal transducer activation [59–62]. Likewise, the PSMAs also exhibit multiple functions involved in various aspects of tumor progression. Knockdown of PSMA7 in myeloid leukemia cell K562 resulted in a marked proliferation inhibition [63]. Although PMSA7 knockdown in colorectal cancer cell line RKO showed no impact on proliferation or cell cycle, depletion of PSMA7 was demonstrated to significantly suppress tumor formation *in vitro* and *in vivo*, as well as inhibit RKO cell invasion and migration [4]. NOD1 is regulated by PSMA7 in a proteasome-dependent manner, and overexpression of PSMA7 inhibits NOD1-mediated colorectal cancer cell apoptosis [64]. The non-small cell lung cancer cell lines A549 and NCI-H460 treated with PSMA1 siRNA showed a loss of the chymotrypsin-like activity of proteasome and a significant decrease in homologous recombination-mediated repair of I-SceI-induced DNA double strand breaks [6]. PSMA3 participates in the ubiquitin-independent degradation of p21, which as a cyclin-dependent kinase inhibitor plays various central roles in cellular processes, by binding and recruiting p21 into proteasome for degradation [65, 66]. Considering the widespread involvement of PSMAs in tumor processes, the potential therapeutic benefits of targeting PSMAs, as well as the limited publications regarding specially PSMA1, PSMA2, PSMA3 and PSMA5, many more studies are needed to further disclose the molecular mechanisms of PSMAs in multiple cancers.

In summary, our study systematically analyzed the mRNA expression levels and prognostic significance of PSMAs in different human cancers. These PSMAs exhibited significant expression differences between tumor and normal tissues in various types of cancer. Moreover, several PSMAs showed great prognostic significance for cancer patients. Future studies are needed to determine the detailed roles of PSMAs in tumor initiation and development, which may strengthen the evidence that PSMAs could be promising therapeutic targets and novel prognostic biomarkers for human carcinomas.

## MATERIALS AND METHODS

### Oncomine database analysis

Oncomine (http://www.oncomine.org), an online microarray database, was utilized to examine the mRNA expression difference of PSMAs between tumor and normal tissues in multiple human cancers. The thresholds were restricted as follows: *p* value: 0.01; fold change: 2; gene rank: 10%; data type: mRNA. For each gene, we performed comparisons by cancer vs. normal analysis. Cancer type, fold change, *t*-test value, *p*-value and sample sizes were obtained from studies that showed statistically significant differences.

### TCGA database analysis

Integrin mRNA HiSeq expression data from the TCGA database involving breast cancer, lung cancer, gastric cancer, colorectal cancer and bladder cancer were downloaded from the Cancer Genomics Browser of University of California Santa Cruz (UCSC) (https://genome-cancer.ucsc.edu/) version: 2015-02-24. Student's *t*-test was performed to investigate the mRNA expression differences between tumor and normal tissues. The boxplots were created by GraphPad software.

### KM Plotter database analysis

We used KM Plotter (http://kmplot.com/analysis/) [67, 68], which contains 4142 breast, 2437 lung, 1648 ovarian and 1065 gastric cancer patients with survival data, to determine the prognostic values of PSMAs in the above four cancers. KM Plotter includs only the Affymetrix HG-U133A, HG-U133 Plus 2.0 and HG-U133A 2.0 microarrays. For each gene symbol, the desired probe ID was identified according to the file of probe sets provided by KM Plotter. Cancer patients were divided into high and low expression group by the median values of mRNA expression, and survival analyses were carried out without follow-up restrictions. Briefly, the desired probe IDs representing the seven genes were separately entered into the database to obtain Kaplan-Meier plots. Number of cases, median values of mRNA expression levels, HRs, 95% CIs and *p*-values were extracted from the KM plotter webpage.

### PrognScan database analysis

For the other kinds of cancers, PrognScan (http://www.abren.net/PrognoScan/) [69], a large database including publicly available microarray datasets with gene expression and survival data for several cancers, was applied to evaluate the prognostic effects of PSMAs. The microarrays and probe IDs selected for each gene were in line with those used in the KM plotter database. The results of the survival analyses were downloaded from PrognScan database.

## SUPPLEMENTARY MATERIALS FIGURES AND TABLES


