# Supplementary material for: The transcription levels and prognostic values of seven proteasome alpha subunits in human cancers
Source: Oncotarget. 2016 Dec 10;8(3):4501–19. doi: 10.18632/oncotarget.13885 (PMC5354849; doi:10.18632/oncotarget.13885)
Supplement: Supplementary file 1 [file oncotarget-08-4501-s001.pdf]

The transcription levels and prognostic values of seven proteasome alpha subunits in human cancers

Supplementary Materials

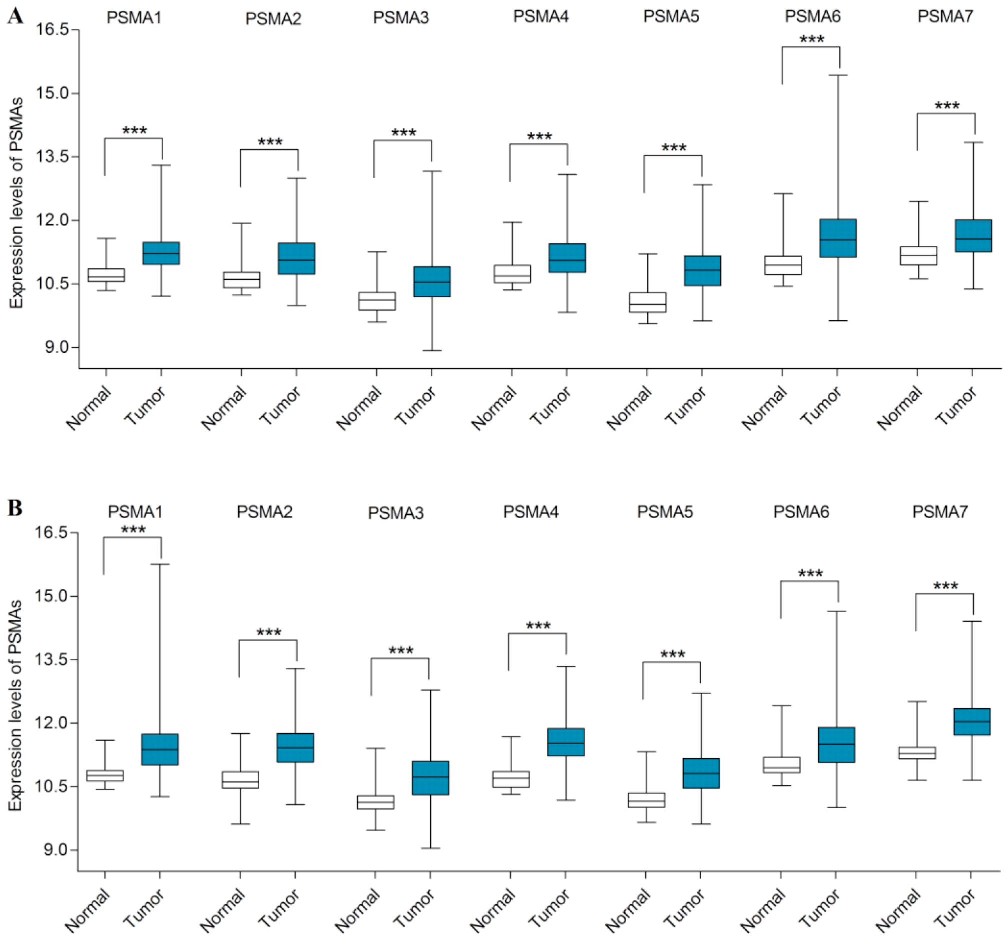

Supplementary Figure S1: mRNA expression levels of PSMAs in lung adenocarcinoma and lung squamous cell carcinoma (TCGA mRNA HiSeq expression data). (**A**) mRNA expression levels of PSMAs were investigated in 511 lung adenocarcinomas and 58 normal tissues. (**B**) mRNA expression levels of PSMAs were investigated in 502 lung squamous cell carcinomas and 51 normal tissues. The line in the middle represents the median value. Statistical differences were examined by two tailed Student's *t*-test. ***$p < 0.001$.

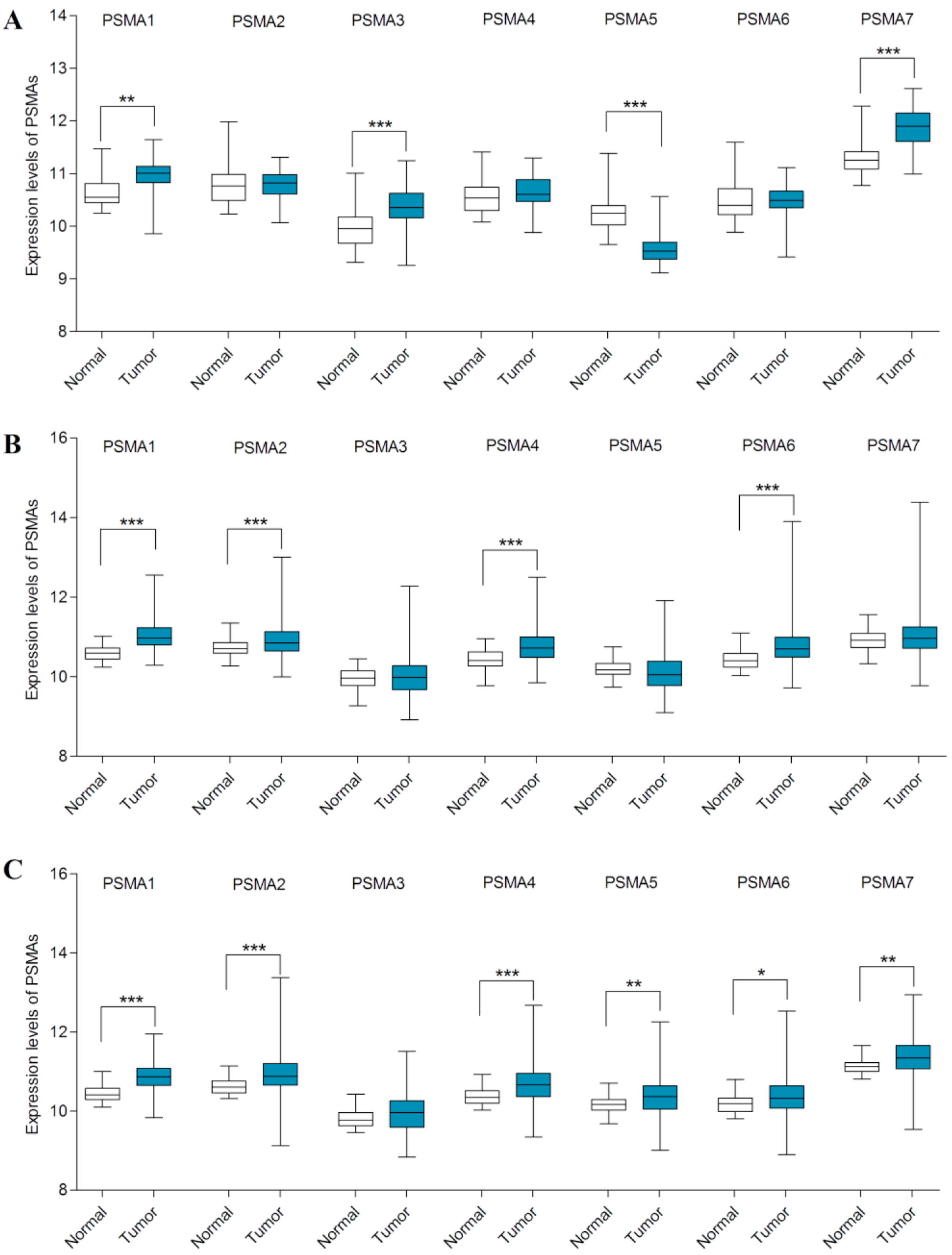

Supplementary Figure S2: mRNA expression levels of PSMAs in three subtypes of kidney cancer (TCGA mRNA HiSeq expression data). (**A**) mRNA expression levels of PSMAs were investigated in 66 kidney chromophobe cell carcinomas and 25 normal tissues. (**B**) mRNA expression levels of PSMAs were investigated in 533 kidney clear cell carcinomas and 72 normal tissues. (C) mRNA expression levels of PSMAs were investigated in 290 kidney papillary cell carcinomas and 32 normal tissues. The line in the middle represents the median value. Statistical differences were examined by two tailed Student's *t*-test. $*p < 0.05$; $**p < 0.01$; $***p < 0.001$.

Supplementary Table S1: The desired probes of PSMAs in KM plotter database

Probe ID	Gene symbol	No. of Probes	Specificity	Coverage	Robust	Overall	Best
211746_x_at	PSMA1	11	0.636	0.667	0.503	0.213	TRUE
201316_at	PSMA2	11	0.636	1.000	0.631	0.402	TRUE
201532_at	PSMA3	11	0.909	1.000	0.419	0.381	TRUE
203396_at	PSMA4	11	1.000	1.000	0.413	0.413	TRUE
230300_at	PSMA5	11	0.909	1.000	0.710	0.646	TRUE
208805_at	PSMA6	11	0.818	1.000	0.569	0.465	TRUE
201114_x_at	PSMA7	11	0.636	0.500	0.442	0.141	TRUE

Supplementary Table S2: The Correlation between PSMAs and survival outcomes in breast cancer patients restricted by intrinsic subtypes. See Supplementary_Table_S2

Supplementary Table S3: The Correlation between PSMAs and survival outcomes in lung cancer patients restricted by histology, tumor stage and tumor grade. See Supplementary_Table_S3

Supplementary Table S4: The Correlation between PSMAs and survival outcomes in gastric cancer patients restricted by tumor stage and HER2 status. See Supplementary_Table_S4
